# A comparison of orthopaedic surgery and internal medicine perceptions of USMLE Step 1 pass/fail scoring

**DOI:** 10.1186/s12909-021-02699-4

**Published:** 2021-05-03

**Authors:** Frederick Mun, Alyssa R. Scott, David Cui, Erik B. Lehman, Seong Ho Jeong, Alia Chisty, Paul J. Juliano, William L. Hennrikus, Eileen F. Hennrikus

**Affiliations:** 1 Penn State College of Medicine, Penn State Milton S. Hershey Medical Center, Hershey, PA USA; 2 Public Health Sciences at Penn State College of Medicine, Penn State Milton S. Hershey Medical Center, Hershey, PA USA; 3 Department of Orthopaedics and Rehabilitation, Yale School of Medicine, Yale New Haven Hospital, New Haven, CT USA; 4 Department of Internal Medicine, Penn State College of Medicine, Penn State Milton S. Hershey Medical Center, Hershey, PA USA; 5 Department of Orthopaedics and Rehabilitation, Penn State College of Medicine, Penn State Milton S. Hershey Medical Center, Hershey, PA USA

**Keywords:** USMLE, Step 1, Pass/fail scoring, Orthopaedic surgery, Internal medicine

## Abstract

**Background:**

United States Medical Licensing Examination Step 1 will transition from numeric grading to pass/fail, sometime after January 2022. The aim of this study was to compare how program directors in orthopaedics and internal medicine perceive a pass/fail Step 1 will impact the residency application process.

**Methods:**

A 27-item survey was distributed through REDCap to 161 U.S. orthopaedic residency program directors and 548 U.S. internal medicine residency program directors. Program director emails were obtained from the American Medical Association’s Fellowship and Residency Electronic Interactive Database.

**Results:**

We received 58 (36.0%) orthopaedic and 125 (22.8%) internal medicine program director responses. The majority of both groups disagree with the change to pass/fail, and felt that the decision was not transparent. Both groups believe that the Step 2 Clinical Knowledge exam and clerkship grades will take on more importance. Compared to internal medicine PDs, orthopaedic PDs were significantly more likely to emphasize research, letters of recommendation from known faculty, Alpha Omega Alpha membership, leadership/extracurricular activities, audition elective rotations, and personal knowledge of the applicant. Both groups believe that allopathic students from less prestigious medical schools, osteopathic students, and international medical graduates will be disadvantaged. Orthopaedic and internal medicine program directors agree that medical schools should adopt a graded pre-clinical curriculum, and that there should be a cap on the number of residency applications a student can submit.

**Conclusion:**

Orthopaedic and internal medicine program directors disagree with the change of Step 1 to pass/fail. They also believe that this transition will make the match process more difficult, and disadvantage students from less highly-regarded medical schools. Both groups will rely more heavily on the Step 2 clinical knowledge exam score, but orthopaedics will place more importance on research, letters of recommendation, Alpha Omega Alpha membership, leadership/extracurricular activities, personal knowledge of the applicant, and audition electives.

**Supplementary Information:**

The online version contains supplementary material available at 10.1186/s12909-021-02699-4.

## Background

The United States Medical Licensing Examinations (USMLE) currently consists of three numerically scored knowledge based exams. On February 12, 2020, the Federation of State Medical Boards (FSMB) and the National Board of Medical Examiners (NBME) announced that the first of these three exams, Step 1, would change from the three-digit numeric score to reporting a pass/fail outcome -- starting sometime after January 2022 [1]. This change occurred after extensive national discussion on potential ways to optimize the transition from undergraduate to graduate medical education [1, 2].

The unintended consequences of a scored Step 1 on medical students and residency programs were widely reported in 2008 by the Committee to Evaluate the USMLE Program (CEUP). CEUP recognized the diverse stakeholders affected by Step 1 score reporting and suggested a need to redesign USMLE [2]. Although a numerically scored Step 1 provided an objective measure for residency programs to assess applicants from different backgrounds and medical schools, the perceived overemphasis on this test was controversial. In March 2019, the Invitational Conference on USMLE Scoring (InCUS), sponsored by the Association of American Medical Colleges (AAMC), American Medical Association (AMA), NBME, FSMB, and Educational Commission for Foreign Medical Graduates (ECFMG), convened to review the USMLE’s practice of numeric score reporting. InCUS concluded that licensing examinations for undergraduate medical students must be reevaluated. FSMB and NBME took into consideration the recommendations from InCUS, and stated that the change to a pass/fail Step 1 would help residency programs to refocus on the main purpose of Step 1 -- to assess medical licensure eligibility [1, 2].

Step 1 is one of the most important factors used to screen applicants by internal medicine (IM) and orthopaedic surgery residency programs [1–3]. Some residency programs also place a high emphasis on other factors, such as grades in required clerkships, research experiences, extracurricular activities, letters of recommendation, and audition rotations. With the absence of a scored Step 1, all residency programs will need to reconsider the factors used to determine which applicants are selected.

The aim of this study was to compare the perceptions among program directors (PDs) in orthopaedics and IM on the change of Step 1 from a graded to a pass/fail exam, and its impact on the residency application process. Some groups have studied PD opinions of the Step 1 pass/fail change; however, to the best of our knowledge, the current study is the first comprehensive survey to directly compare the impact on medicine and a surgical subspecialty [4–12]. We chose to study IM PDs because IM has historically been a popular specialty that has had the most number of total applicants. We decided to study Orthopaedic PDs because orthopaedics has historically received some of the highest number of applicants per residency position [3, 13]. By directly comparing medicine and a surgical subspecialty, we hoped to gain insight into the diverse priorities and perceptions among PDs.

## Methods

This study was exempted by the College of Medicine’s Institutional Review Board. We obtained publicly available contact information for PDs at all active Accreditation Council for Graduate Medical Education (ACGME) orthopaedic surgery and IM residency programs through the American Medical Association’s Fellowship and Residency Electronic Interactive Database (FREIDA Online). We identified and contacted 197 orthopaedic surgery PDs and 554 internal medicine PDs. A survey invitation email with the appropriate informed consent information was sent to each PD. Submission of the survey indicated the respondents’ consent to participate. All responses were collected anonymously.

This multi-center, cross-sectional research survey was developed, housed, and distributed through REDCap Data Capture. The survey was developed using criteria analyzed in the 2018 National Resident Matching Program (NRMP) Program Director survey, which investigated the factors involved in selecting applicants to interview [3]. Our survey questions used single-answer multiple-choice, multiple-answer multiple-choice, and a Likert scale ranging from 0 (strongly disagree or least important) to 4 (strongly agree or most important) formats. Prior to distribution, the questions were pretested and tested with subsets of respondents resulting in a 27-item survey. No funding was utilized.

The orthopaedic electronic survey was distributed on April 17, 2020. A follow-up was emailed 2 weeks after the initial submission. The survey was closed on May 5, 2020. The IM electronic survey was distributed on July 8, 2020. A follow-up was emailed 2 weeks after the initial submission. The survey was closed on August 5, 2020.

Orthopaedic and IM PD responses were summarized with descriptive statistics and percentages prior to any analysis to check their distributions. Given that the outcome variables were ordinal variables on a Likert scale of agreement or importance ranging from 0 to 4, we used a Wilcoxon Rank Sum test to compare the medians of the survey responses of orthopaedic and IM PDs. For binary outcomes, we used a Chi-Square test to look for any differences between the groups. Statistical significance was set at *p* < 0.05, and all analyses were performed using SAS version 9.4 (SAS Institute, Cary, NC).

To ensure a more robust analysis, the authors combined those who scored 0 or 1 on the Likert scale into one category as “disagree” or “less important.” Those who scored 3 or 4 on the Likert scale were combined as “agree” or “more important.”

## Results

Of the 197 orthopaedic programs, we identified contact information for 161 to invite. Of those, 58 responded for a response rate of 36.0%. Of the 554 total IM programs, we identified contact information for 548 to invite. Of those, 125 IM PDs responded for a response rate of 22.8%. The Likert scale data, including median Likert scores, interquartile range, and *p*-values, are shown in Table 1. The single-answer multiple-choice and multiple-answer multiple-choice data, including number of respondents for each survey question, and *p*-values, are shown in Table 2.

**Table 1:** A Comparison of Orthopaedic and Internal Medicine Program Directors’ Perceptions of the USMLE Step 1 Pass/Fail Transition: Likert Scale Responses

	Orthopaedics-median Likert score (IQR)	Internal Medicine-median Likert score (IQR)	*p*-value
***How do orthopaedic and internal medicine program directors feel about the pass/fail Step 1?*** **0 = strongly disagree, 1 = disagree, 2 = neutral, 3 = agree, 4 = strongly agree**			
I agree with the change to pass/fail Step 1	0.0 (1.0)	1.0 (2.0)	0.313
The decision to transition to pass/fail Step 1 was transparent and adequatelyinvolved all stakeholders	1.0 (1.0)	0.0 (1.0)	0.412
A graded Step 1 adequately measured the ability of an applicant to succeed	2.0 (1.0)	2.0 (2.0)	0.574
***How will a pass/fail Step 1 impact orthopaedic and internal medicine residency programs?*** **0 = strongly disagree, 1 = disagree, 2 = neutral, 3 = agree, 4 = strongly agree**			
A pass/fail Step 1 will make the match process fair and meritocratic	2.0 (2.0)	1.0 (2.0)	0.028*
A pass/fail Step 1 will help to create better future physicians	2.0 (2.0)	1.0 (2.0)	0.211
***How will the pass/fail change impact the importance of the factors reviewed by orthopaedic and internal medicine residency programs when assessing applicants?*** **0 = significantly less important, 1 = less important, 2 = no change, 3 = more important,4 = significantly more important**			
Step 1 exam result	0.0 (1.0)	1.0 (2.0)	< 0.001*
Step 2 CK	4.0 (1.0)	4.0 (1.0)	0.159
Step 2 CS	2.0 (1.0)	2.0 (1.0)	0.423
Grades in required clerkships	3.0 (1.0)	3.0 (1.0)	0.060
Research experience	2.5 (1.0)	2.0 (0.0)	< 0.001*
Letters of recommendation from orthopaedic/internal medicine faculty thatprogram directors know	3.0 (1.0)	3.0 (1.0)	< 0.001*
Letters of recommendation from orthopaedic/internal medicine faculty thatprogram directors do not know	3.0 (1.0)	2.0 (1.0)	0.103
Letters of recommendation from faculty not within specialty	2.0 (0.0)	2.0 (0.0)	0.350
Personal statement	2.0 (0.0)	2.0 (0.0)	0.665
Medical student performance evaluations (MSPE)/Dean’s letter	2.0 (1.0)	3.0 (1.0)	0.120
Alpha Omega Alpha (AOA) membership	3.0 (2.0)	2.0 (1.0)	< 0.001*
Gold Humanism Society membership	2.0 (1.0)	1.0 (1.0)	0.069
Leadership/extracurriculars	3.0 (1.0)	2.0 (1.0)	< 0.001*
Personal knowledge of applicant	4.0 (1.0)	3.0 (2.0)	< 0.001*
Audition electives within department	4.0 (1.0)	3.0 (1.0)	< 0.001*
***How will various applicant groups be affected by the change to a pass/fail Step 1?*** **0 = greatly disadvantaged, 1 = disadvantaged, 2 = neutral, 3 = advantaged, 4 = greatlyadvantaged**			
All MD students	2.0 (1.0)	2.0 (1.)	0.098
MD students who attend a highly-regarded medical schools	3.0 (2.0)	3.0 (1.0)	0.599
MD students who do not attend a highly-regarded medical school	1.0 (1.0)	1.0 (1.0)	0.011*
DO students	1.0 (2.0)	1.0 (1.0)	0.001*
International medical graduates (IMGs)	1.0 (2.0)	1.0 (1.0)	0.146

Table 1 shows the distribution of responses, from all U.S. orthopaedic and internal medicine residency program directors, to our survey, specifically the responses to questions using a Likert scale. The bolded and italicized text in the table are the main questions asked in our survey, with the conditions of the Likert scale included. The median Likert scores were compared between orthopaedic and internal medicine program director. An asterisk (*) indicates statistical significance (*p* < 0.05). IQR indicates the interquartile range

**Table 2:** A Comparison of Orthopaedic and Internal Medicine Program Directors’ Perceptions of the USMLE Step 1 Pass/Fail Transition: Single-answer and Multiple-answer Multiple-choice Responses

	Orthopaedics- N (%)	Internal Medicine-N (%)	*p*-value
***How will changing to a pass/fail Step 1 affect medical students interested in orthopaedics and internal medicine?***			
Allow students to focus more on learning medicine rather than studying for Step 1	13 (22.4%)	32 (25.6%)	0.641
Encourage more research experiences	23 (39.7%)	12 (9.6%)	< 0.001*
Encourage more leadership/extracurriculars	16 (27.6%)	23 (18.4%)	0.158
Allow students to pursue more hobbies/self-development	6 (10.3%)	27 (21.6%)	0.065
Encourage students to attend more audition electives	34 (58.6%)	36 (28.8%)	< 0.001*
Encourage applicants to apply to more residency programs	39 (67.2%)	71 (56.8%)	0.180
Encourage applicants to apply to other specialties in addition to their primary specialty of interest	27 (46.6%)	49 (39.2%)	0.348
***What are the future implications on residency applications and medical education?***			
With the change to pass/fail Step 1, medical schools should adopt a graded pre-clinical curriculum	37 (63.8)	65 (52.0)	0.810
With the change to pass/fail Step 1, there should be a cap on the number of residency applications a medical student can submit	42 (72.4)	69 (55.2)	0.198

Table 2 shows the distribution of responses, from all U.S. orthopaedic and internal medicine residency program directors, to our survey, specifically the responses to single-answer and multiple-answer multiple-choice questions. The bolded and italicized text in the table are the main questions asked in our survey. The table shows N (%) of the orthopaedic and internal medicine PDs who answered “yes” to the listed statements. An asterisk (*) indicates statistical significance (*p* < 0.05)

### How do internal medicine and orthopaedic program directors feel about the pass/fail Step 1?

The majority of orthopaedic (82.8%) and IM (74.4%) PDs disagree with the change to pass/fail Step 1. Additionally, 86.2% of orthopaedic and 79.2% of IM PDs disagree that the decision to transition to pass/fail Step 1 was transparent. Only 43.1% of orthopaedic and 39.2% of IM PDs agree that a graded Step 1 measures the ability of applicants to succeed in their respective fields. Orthopaedic and IM PDs both equally disagree with the change to pass/fail Step 1 (*p* = 0.313) and agree that a graded Step 1 does not adequately measure the ability of an applicant to succeed in residency (*p* = 0.574).

### How will a pass/fail Step 1 impact internal medicine and orthopaedic residency programs?

Few orthopaedic (10.4%) and IM (20.7%) PDs agree that a pass/fail Step 1 will help to create better future physicians. IM PDs felt more strongly than Orthopaedic PDs that the change to pass/fail Step 1 will make the match process less fair and meritocratic (*p* = 0.028).

### How will the pass/fail change impact the importance of the factors reviewed by internal medicine and orthopaedic residency programs when assessing applicants?

The majority of orthopaedic (89.7%) and IM (69.6%) PDs believe that Step 1 will become less important when it transitions to pass/fail, and 96.9% of orthopaedic and 88.0% of IM PDs agree that Step 2 Clinical Knowledge exam (CK) score will be more important. Additionally, 77.6% of orthopaedic and 67.2% of IM PDs say grades in required clerkships will be more important. The majority of both orthopaedic and IM PDs believe that personal knowledge of the applicant and an audition elective will be more important.

Compared to IM PDs, orthopaedic PDs were significantly more likely to say the following components will be of greater importance after the change to pass/fail Step 1: research experience (*p* < 0.001); letters of recommendation from faculty they know (*p* < 0.0.001); Alpha Omega Alpha (AOA) membership (*p* < 0.001); leadership/extracurricular activities (*p* < 0.001); personal knowledge of the applicant (*p* < 0.001) and audition electives (*p* < 0.001).

### How will various applicant groups be affected by the change to a pass/fail Step 1?

The majority of orthopaedic and IM PDs believe that allopathic (MD) students not attending highly-regarded medical schools, osteopathic (DO) students, and international medical graduates (IMGs) will be disadvantaged by the change of Step 1 to pass/fail. Compared to IM PDs, orthopaedic PDs were more likely to believe that MD students who do not attend highly-regarded medical schools (*p* = 0.011) and DO students (*p* = 0.001) will be disadvantaged in the match processes due to the Step 1 pass/fail transition.

### How will changing to a pass/fail Step 1 affect medical students interested in internal medicine and orthopaedics?

Orthopaedic and IM PDs both have low expectations (< 30%) that the change to a pass/fail Step 1 will allow students to focus more on learning medicine rather than studying for the Step exam, to seek out more leadership and extracurricular activities, or to pursue more hobbies and self-development. Orthopaedic PDs (39.7%), compared to IM PDs (9.6%), were more likely to believe a pass/fail Step 1 will encourage applicants to pursue more research experiences (*p* < 0.001). Less than 10% of IM PDs thought the pass/fail change would encourage more research experience. Compared to IM PDs (28.8%), orthopaedic PDs (58.6%) were more likely to think that the Step 1 pass/fail change would encourage students to attend more audition electives (*p* < 0.001).

### What are the future implications on residency applications and medical education?

With the change to pass/fail Step 1, a majority of orthopaedic and IM PDs agree that medical schools should adopt a graded pre-clinical curriculum, and that there should be a cap on the number of residency applications an applicant can submit.

## Discussion

The present study shows that the majority of orthopaedic and IM PDs do not support the Step 1 pass/fail change. Both orthopaedic and IM PDs believe Step 2 CK will be significantly more important. After the pass/fail change, orthopaedic PDs are significantly more likely than IM PDs to assign greater value to research experience, letters of recommendation from faculty they know, AOA membership, leadership/extracurricular activities, personal knowledge of the applicant, and audition electives. Both orthopaedic and IM PDs believe students who attend highly-regarded medical schools will be advantaged by the move to make Step 1 pass/fail. However, more orthopaedic PDs believe DO students and MD students attending less prestigious medical schools will be disadvantaged, while more IM PDs believe IMGs will be disadvantaged.

In December 2018, USMLE contacted major stakeholders, including orthopaedic and IM PDs, for public comment regarding USMLE scoring, and received over 37,000 responses. In March 2019, InCUS was sponsored by the AAMC, AMA, NBME, FSMB, and ECFMG, to discuss the issue of USMLE scores, and to explore potential avenues for improving the current scoring system. Following InCUS, USMLE presented their findings to the AMA Council on Medical Education, and made their preliminary findings publicly available on their website. Before officially announcing the scoring transition in February 2020, USMLE provided updates via their website, social media outlets, podcasts, and presentations at national meetings [1, 2]. Despite these steps, in the present study, a majority of orthopaedic and IM PDs believed that the transition to pass/fail was not transparent, suggesting that USMLE, and various stakeholders in medical education, such as PDs, may need to assess ways to improve communication with each other.

Currently, standardized examinations play a major role in evaluating residency applicants across specialties, with Step 1 often being considered the most important test [1–3]. In the 2020 NRMP Survey, PDs across all specialties were asked to rank the factors used to interview and rate applicants on a scale of 1 (not at all important) to 5 (very important). When selecting which applicants to interview, orthopaedic and IM PDs provided high ratings, over 4/5, for Step 1. Furthermore, over 90% of orthopaedic and IM PDs cited Step 1 as an important factor for screening applicants for an interview [3].

There is minimal evidence supporting a scored Step 1’s ability to screen for successful residents [14–18]. Even in our survey, the majority of both orthopaedic and IM PDs felt that a graded Step 1 did not adequately measure the ability of an applicant to succeed in residency. Yet, the majority of orthopaedic and IM PDs also disagree with the pass/fail change, suggesting that Step 1 functions mainly as a convenient screening metric. A majority also support application caps, which may suggest that support for the numeric Step 1 is primarily necessitated by the burden of programs receiving high numbers of applications [1, 2]. Despite the limited predictive value of standardized tests for future success, both orthopaedics and IM have used Step 1 as a screening measure for applicants due to the lack of additional objective criteria [3]. Historically, both orthopaedics and IM have emphasized standardized test scores. According to the NRMP, U.S. MD seniors applying to orthopaedics had an average Step 1 score of 248 and a Step 2 CK score of 255. U.S. MD seniors applying to IM had an average Step 1 score of 235 and a Step 2 CK score of 248 [19]. In our study, the majority of orthopaedic and IM PDs report that Step 2 CK scores will be more important.

The results of our study also suggest that these specialties may place a greater emphasis on factors outside of standardized exam scores, as both orthopaedic and IM PDs believe grades in required clerkships, personal knowledge of the applicant, and audition electives will now be more important. Additionally, orthopaedic PDs are more likely than IM PDs to say research experience, AOA membership, letters of recommendation from faculty they know, leadership/extracurricular activities, personal knowledge of the applicant, and audition electives will be more important. Based on our analysis, orthopaedic PDs are also significantly more likely to believe a pass/fail Step 1 will encourage applicants to pursue more research experiences and to say it will encourage students to attend more audition electives. Recently, many orthopaedic programs have been publicizing holistic changes in their residency selection criteria on social media. For instance, the University of Missouri - Kansas City Orthopaedic Surgery Residency program announced on Twitter that they would no longer be considering Step 1 and Step 2 CK scores. They plan on considering other factors like personal statement, recommendation letters, dean’s letter, research, audition rotations, grades in required clerkships, and interest in their program [20]. This is one example of an orthopaedic program moving toward a more holistic application review. A possible positive consequence of this move towards a more holistic process is that students from nontraditional, underrepresented, or underserved backgrounds may have more opportunities to be selected for interviews [21].

Interestingly, in the 2020 Match, U.S. MD seniors applying to orthopaedics had an average of 14.3 abstracts/presentations/publications, 8 volunteer experiences, and 40.3% were part of AOA; those applying to IM had an average of 6.2 abstracts/presentations/publications, 7.3 volunteer experiences, and 17.4% were part of AOA [19]. This difference may be due to the more competitive match process in orthopaedics. In 2020, U.S. MD seniors applying to IM had a match rate 97.1%, while orthopaedics had a match rate of 79.7% [19].

This work found that 83.2% of IM PDs believe IMGs will be disadvantaged by the change to pass/fail Step 1, while only 58.6% of orthopaedic PDs believe IMGs will be disadvantaged. This difference may be related to the lack of IMGs in orthopaedics, and the prevalence of IMGs in IM residencies. In 2019, only 1.5% of orthopaedic residents were IMGs, while 38.8% of IM residents were IMGs [22]. IMGs frequently rely on Step 1 scores to help them stand out when applying to US residency programs. Many residency programs often use Step 1 scores to quickly sift through the large volume of IMG applicants [23–25]. IM PDs will have to consider new criteria by which to evaluate IMGs, while orthopaedic PDs will likely not face this challenge to the same extent, as IMGs make up such a small portion of those entering the field of orthopaedic surgery.

The present study demonstrated that orthopaedic PDs are more likely to say DOs will be disadvantaged by the change to a pass/fail Step 1. When applying for residency, DO applicants may face additional challenges compared to MD students [25, 26]. This may be due to a variety of factors, including limited research opportunities for students at DO schools compared to students at MD schools, as most US osteopathic medical schools are typically not associated with large academic institutions [26].

A limitation of this study was that we were unable to obtain responses from all orthopaedic and IM residency PDs. Our study may be limited by sampling bias and non-response bias. Respondents and non-respondents may have differed systematically, as PDs who feel strongly about this topic may have been more inclined to respond. However, we believe our data demonstrates responses from a substantial portion of PDs in orthopaedics (58 PDs, 36.0%) and in IM (125 PDs, 22.8%), with representation from all four regions of the United States (West, Midwest, South, and Northeast), community/academic programs, and private/public programs.

Another limitation of this study is that some contact information provided by FREIDA Online did not belong to the PD, but instead the program’s coordinator or administrative assistant. Our email requested program coordinators and administrative assistants to send the survey to PD.

## Conclusions

In conclusion, the majority of orthopaedic and IM PDs do not support the Step 1 pass/fail change. Both groups will rely more heavily on Step 2 CK. Orthopaedic PDs are significantly more likely to value research experience, letters of recommendation from faculty they know, AOA membership, leadership/extracurricular activity, personal knowledge of the applicant, and audition electives.

## Supplementary Information

**Additional file 1.** Internal Medicine and Orthopaedic Surgery Program Director Step 1 Pass/Fail Perceptions Survey.

## Data Availability

The datasets generated and/or analyzed during the current study are not publicly available because the survey was conducted independently at our institution, but are available from the corresponding author on reasonable request.

## Abbreviations

USMLE: United States Medical Licensing Examinations
FSMB: Federation of State Medical Boards
NBME: National Board of Medical Examiners
CEUP: Committee to Evaluate the USMLE Program
InCUS: Invitational Conference on USMLE Scoring
AAMC: Association of American Medical Colleges
AMA: American Medical Association
ECFMG: Educational Commission for Foreign Medical Graduates
ACGME: Accreditation Council for Graduate Medical Education
IM: Internal Medicine
PD: Program Director
FREIDA Online: Fellowship and Residency Electronic Interactive Database
NRMP: National Resident Matching Program
CK: Clinical Knowledge exam
AOA: Alpha Omega Alpha
IMG: International Medical Graduates
COMLEX: Comprehensive Osteopathic Medical Licensing Examination
