# Correction to: A comparison of orthopaedic surgery and internal medicine perceptions of USMLE Step 1 pass/fail scoring

**DOI:** 10.1186/s12909-021-02988-y

**Published:** 2021-10-27

**Authors:** Frederick Mun, Alyssa R. Scott, David Cui, Erik B. Lehman, Seongho Jeong, Alia Chisty, Paul J. Juliano, William L. Hennrikus, Eileen F. Hennrikus

**Affiliations:** 1 Penn State College of Medicine, Penn State Milton S. Hershey Medical Center, Hershey, PA USA; 2 Public Health Sciences at Penn State College of Medicine, Penn State Milton S. Hershey Medical Center, Hershey, PA USA; 3 Department of Orthopaedics and Rehabilitation, Yale School of Medicine, Yale New Haven Hospital, New Haven, CT USA; 4 Department of Internal Medicine, Penn State College of Medicine, Penn State Milton S. Hershey Medical Center, Hershey, PA USA; 5 Department of Orthopaedics and Rehabilitation, Penn State College of Medicine, Penn State Milton S. Hershey Medical Center, Hershey, PA USA

**Correction to: BMC Med Educ 21, 255 (2021)**

**https://doi.org/10.1186/s12909-021-02699-4**

Following publication of the original article [1], the authors identified an error in the author name of Seongho Jeong.

The incorrect author name is: Seong Ho Jeong

The correct author name is: Seongho Jeong

The author group has been updated above and the original article [1] has been corrected.

## References

[1] Mun (2021). A comparison of orthopaedic surgery and internal medicine perceptions of USMLE Step 1 pass/fail scoring. BMC Med Educ.

