# Supplementary material for: A comparison of orthopaedic surgery and internal medicine perceptions of USMLE Step 1 pass/fail scoring
Source: BMC Med Educ. 2021 May 3;21:255. doi: 10.1186/s12909-021-02699-4 (PMC8091716; doi:10.1186/s12909-021-02699-4)
Supplement: Supplementary file 1 — Additional file 1. Internal Medicine and Orthopaedic Surgery Program Director Step 1 Pass/Fail Perceptions Survey. [file 12909_2021_2699_MOESM1_ESM.docx]

Supplement 1: Internal Medicine and Orthopaedic Surgery Program Director Step 1 Pass/Fail Perceptions Survey

1. How do you feel about the following statements?

|  | Strongly disagree (0) | Disagree (1) | Neutral (2) | Agree (3) | Strongly Agree (4) |
| --- | --- | --- | --- | --- | --- |
| I agree with the change to a pass/fail Step 1. |  |  |  |  |  |
| The decision to transition to pass/fail Step 1 was transparent and adequately involved all stakeholders. |  |  |  |  |  |
| A graded Step 1 adequately measured the ability of an applicant to succeed. |  |  |  |  |  |

2. How will a pass/fail Step 1 impact the following?

|  | Strongly disagree (0) | Disagree (1) | Neutral (2) | Agree (3) | Strongly Agree (4) |
| --- | --- | --- | --- | --- | --- |
| A pass/fail Step 1 will make the match process fair and meritocratic |  |  |  |  |  |
| A pass/fail Step 1 will help to create better future physicians |  |  |  |  |  |

3. With the change to pass/fail Step 1, how will the impact on resident selection of each of the following factors of a student’s application change at your institution?

|  | Significantly less important (0) | Less important (1) | No change (2) | More important (3) | Significantly more important (4) |
| --- | --- | --- | --- | --- | --- |
| Step 1 exam result |  |  |  |  |  |
| Step 2 CK |  |  |  |  |  |
| Step 2 CS |  |  |  |  |  |
| Grades in required clerkships |  |  |  |  |  |
| Research experience |  |  |  |  |  |
| Letters of recommendation from orthopaedic/internal medicine faculty that program directors know |  |  |  |  |  |
| Letters of recommendation from orthopaedic/internal medicine faculty that program directors do not know |  |  |  |  |  |
| Letters of recommendation from faculty not within specialty |  |  |  |  |  |
| Personal statement |  |  |  |  |  |
| Medical student performance evaluations (MSPE)/Dean’s letter |  |  |  |  |  |
| Alpha Omega Alpha (AOA) membership |  |  |  |  |  |
| Gold Humanism Society membership |  |  |  |  |  |
| Leadership/extracurriculars |  |  |  |  |  |
| Personal knowledge of applicant |  |  |  |  |  |
| Audition electives within department |  |  |  |  |  |

4. How will various applicant groups be affected by the change to a pass/fail Step 1?

|  | Greatly disadvantaged (0) | Disadvantaged (1) | Neutral (2) | Advantaged (3) | Greatly advantaged (4) |
| --- | --- | --- | --- | --- | --- |
| All MD students |  |  |  |  |  |
| MD students who attend a highly-regarded medical schools |  |  |  |  |  |
| MD students who do not attend a highly-regarded medical school |  |  |  |  |  |
| DO students |  |  |  |  |  |
| International medical graduates (IMGs) |  |  |  |  |  |

5. How will changing to a pass/fail Step 1 affect medical students interested in your specialty? Select all that apply:

| Allow students to focus more on learning medicine rather than studying for Step 1 |  |
| --- | --- |
| Encourage more research experiences |  |
| Encourage more leadership/extracurriculars |  |
| Allow students to pursue more hobbies/self-development |  |
| Encourage students to attend more audition electives |  |
| Encourage applicants to apply to more residency programs |  |
| Encourage applicants to apply to other specialties in addition to their primary specialty of interest |  |

6. In 2019, 66% of allopathic (MD) medical schools have a pass/fail pre-clinical curriculum. With the pass/fail Step 1, do you believe schools should adopt a graded pre-clinical curriculum?

| Yes |  |
| --- | --- |
| No |  |
| Unsure |  |

7. Should there be a cap on the number of residency applications a medical student can submit?

| Yes |  |
| --- | --- |
| No |  |
| Unsure |  |
